# No differences at return to sport in psychological profiles and results of muscle function tests between females with and without a second ipsilateral or contralateral ACL injury after ACL reconstruction

**DOI:** 10.1186/s13102-026-01573-4

**Published:** 2026-02-07

**Authors:** Balint Zsidai, Jakob Lindskog, Rebecca Hamrin Senorski, Roland Thomeé, Axel Sundberg, Johan Högberg, Ramana Piussi

**Affiliations:** 1 Sahlgrenska Sports Medicine Center, Gothenburg University, Box 100, Gothenburg, 40530 Sweden; 2 Unit of Physiotherapy, Department of Health and Rehabilitation, Institute of Neuroscience and Physiology, Sahlgrenska Academy, University of Gothenburg, Box 455, Gothenburg, SE-405 30 Sweden; 3 Department of Orthopaedics, Institute of Clinical Sciences, Sahlgrenska Academy, University of Gothenburg, Gothenburg, Sweden; 4 Department of Orthopedics, Skåne University Hospital, Malmö/Lund, Sweden

**Keywords:** Muscle function, Patient-reported outcomes, Evaluation, Knee, Return to sport

## Abstract

**Background/objective:**

To determine whether psychological response, self-reported knee function and muscle function at return to sport differ between female patients who returned to sport within 12 months after ACL reconstruction (ACL-R) and either did or did not sustain a second ACL injury within 24 months after ACL-R.

**Methods:**

This cohort study included female patients from two Swedish registries, who were between 16 and 40 years old, treated with primary ACL-R between 2014 and 2020, who returned to sport (Tegner ≥ 6) within 12 months from ACL-R, and had at least a 24-month follow-up. The primary focus was comparison of psychological and muscle function profiles at return to sport. Unadjusted comparisons of patient-reported outcomes and muscle function tests at the time of RTS were performed between patients who did and did not sustain a second ipsilateral or contralateral ACL injury during the follow-up period.

**Results:**

A total of 344 female patients were included; 40 (11%) sustained a second ACL injury. No between-groups differences were observed in psychological response or muscle function. For self-reported knee function, females with a second ACL injury reported less severe symptoms (83.8 vs. 78.5, mean difference 5.33, 95% CI 0.68–10.31, ES = 0.37, *p* = 0.024) compared to females who did not sustain a second ACL injury.

**Conclusion:**

There were no differences in psychological response or muscle function between female patients with or without a second ACL injury within 24 months when RTS was achieved within 12 months after primary ACL-R. However, females with a second injury reported slightly less severe symptoms at RTS. Clinically, these findings indicate that the absence of symptoms or psychological concerns at return to sport should not be interpreted as low reinjury risk, and that return-to-sport decisions in female patients may require assessment approaches beyond single time-point patient-reported outcomes or muscle function tests.

## Background

Over the past decades, increasing female sports participation has been accompanied by a rise in the incidence of surgically treated anterior cruciate ligament (ACL) injuries in the athletic population [1, 2]. Several studies have investigated the impact of sex-differences on ACL injury predisposition, reinjury rate, and treatment outcomes [3–5]. Among reported non-modifiable factors associated with ACL injury incidence, females can have a 3- to 6-fold increase in injury risk, as compared to males, for individuals who participate in cutting, jumping or pivoting sports [6, 7]. A discrepancy in self-reported and functional outcomes exists between female and male patients at various follow-up intervals after ACL reconstruction (ACL-R), where females show greater persistent knee laxity, inferior patient-reported outcomes, and greater risk of revision of the ACL-R [5]. In the Swedish population, ACL revision or a contralateral ACL-R were performed in 22% of 15–18 year old female football players over a 5-year follow-up period after a primary ACL-R, which highlights the susceptibility of young female athletes for a second ACL injury [8]. There is a noteworthy 33.7% increase in the risk of a contralateral second ACL injury among females compared to males [9]. 

Return to sport (RTS) is an important outcome in the athletic population who undergo ACL-R [10]. Females have demonstrated an inferior rate of RTS compared to males during the first year after ACL-R, where RTS rate was 39% vs. 52% in males for those aged ≤ 25, and 18% vs. 37%, for those aged 26–35 [11, 12]. Several potential mechanisms may contribute to this discrepancy between females and males, such as differences in the recovery of self-reported knee function, muscle strength, and psychological response towards RTS [13, 14]. Also, muscle strength and psychological response have previously been linked to a second ACL injury risk in both males and females after ACL-R [15, 16]. 

The between-sex discrepancy in self-reported knee function, recovery of muscle strength, and psychological response towards RTS after ACL-R warrants further investigation to determine sex-specific factors that contribute to the increased ACL reinjury risk in active females.

The aim of this study was to determine whether psychological response, self-reported knee function, and muscle function at return to sport differ between females who returned to sport within 12 months after ACL-R and either did or did not sustain a second ACL injury within 24 months after ACL-R.

## Materials and methods

This study adheres to the REporting of studies Conducted using Observational Routinely collected Data (RECORD) statement for routinely collected data and follows the STrengthening the Reporting of OBservational studies in Epidemiology (STROBE) guidelines for observational studies [17, 18]. 

This study was based on prospectively collected data from two registries – the Swedish Knee Ligament Registry (SKLR) and Project ACL. Participation in the registries is voluntary for all parties (patients, physical therapists and surgeons). All parties can withdraw from participation at any time without reason. For the present study, information for the patients included was obtained by linking individual-level data across the SKLR and Project ACL.

The SKLR was established in 2005 and covers over 90% of all ACL reconstructions performed annually in Sweden [19]. The registry consists of two sections: a patient-reported section, and a surgeon-reported section. In the patient section, patients answer web-based questionnaires for self-reported knee function, as well as the physical activity performed at time of sustaining the ACL injury. In the surgeon section, information about the surgical details for each individual is entered via a standardized form by the surgeon at the date of surgery, which includes, but is not limited to, specifications of the ACL-R procedure, and concomitant injuries. Details of the data collection have been described previously [8, 20]. In this study, data from the SKLR was used to identify demographical data and surgical information about the ACL-R, such as concomitant injuries.

Project ACL is a local rehabilitation specific registry, which aims to improve the care of patients who suffer an ACL injury, independent of treatment [21]. Project ACL provides standardized, repeated evaluations of both self-reported and objective outcomes, including muscle function tests and patient-reported outcomes (PROs). The results from evaluations are made available to both patients and their health caregivers. Evaluations are scheduled at 10 weeks, 4, 8, 12, 18 and 24 months, and then every fifth year after the baseline event, that is, ACL injury or ACL-R. Informed consent is collected prior to participation. This research was conducted in accordance with the Declaration of Helsinki, and Project ACL has ethical approval from the Regional Ethical Review Board in Gothenburg, Sweden (registration number: 265 − 13 and T023-17), and from the Swedish Ethical Review Authority (registration number: 2024-08724-01 and 2020–02501). In Project ACL, participation is voluntary and written informed consent to participate and to allow use of de-identified registry data for research is obtained from all participants prior to registration. Accordingly, informed consent was obtained from all individual participants included in this study. Project ACL registry has previously been described in detail [21, 22]. 

Using date of birth and date of surgery, data for patients can be merged between the two registries.

### Patients included

Female patients recorded in the SKLR who underwent primary ACL reconstruction were available for inclusion. Eligible patients were 16–40 years at surgery and had surgery between 2014 and 2020 and had a pre-injury Tegner [23] level above 6. Patients who RTS later than 12 months after ACL-R, suffered a second ipsilateral or contralateral ACL injury later than 24 months after ACL-R, or could not be matched across registries were excluded. The 12-month limit for RTS was chosen because many patients RTS just before this time point [24], to ensure that the PROs and muscle function tests were collected close enough to the second injury to reflect the patients’ status before reinjury, and to create a homogenous group. A maximum follow-up of 24 months after ACL-R was set to reduce the risk of misclassifying a second ACL injury that occurred long after the RTS testing and was unlikely to be related to the tested status at RTS.

### Variables

Patients in Project ACL are assessed via validated PROs and muscle function tests that evaluate unilateral muscle strength performance in isokinetic knee extension and flexion, and hop performance in vertical hop, hop for distance, and side hop. All tests are supervised by trained rehabilitation staff. Patients perform a warm-up according to a standardized procedure previously published [25], that consists of 10 min on a stationary bike and sub-maximal trials on each test. Isokinetic strength test is performed at an angular velocity of 90° per second (Biodex System 4; Biodex Medical System, Shirley, NY, USA). The Biodex dynamometer has been reported to have good reliability (intraclass correlation coefficient [ICC] = 0.95) to test muscle strength [26]. Three maximum trials with 40 s of rest between trials are performed, and peak torque in Newton meter (Nm) is registered in Project ACL’s database.

After strength tests, patients perform hop tests as the second part of the muscle function assessment [27]. During hop tests, patients are required to hop with hands held behind their back. Vertical hop was recorded as flight time from take-off to landing and converted into centimeters (cm) (Muscle lab, Ergotest Technology, Oslo, Norway). In the hop for distance, distance is measured in cm from toe at take-off to heel at landing. Patients are required to perform a stable landing, without letting hands go from behind their back, without using opposite leg for support, and without movement in the landing foot. In the vertical hop and the hop for distance, three maximum effort trials per leg are performed. The 30 s side hop test is performed over two lines 40 cm apart, where number of hops are recorded. In the side hop, one trial per leg is allowed. The best result from the vertical hop and the hop for distance, as well as the result from the side hop test are recorded in Project ACL’s database. Results from the muscle function tests in this study are presented as limb symmetry index (LSI), which is calculated by dividing the result of the injured leg with the result of the non-injured leg and multiplying by 100.

As for the PROs, this study includes the ACL-Return to Sport after Injury scale (ACL-RSI), the Knee injury and Osteoarthritis Outcome Score (KOOS), the 18-item version of the knee self-efficacy scale (K-SES_18_) and the Tegner activity scale (Tegner).

The ACL-RSI aims to measure confidence, emotion and risk appraisal towards RTS after an ACL injury [28]. The ACL-RSI consists of 12-items, where patients report emotion, confidence and risk appraisal from 1 i.e., extremely negative, to 10 i.e., extremely positive response. A total score is calculated by adding response to each item, and ranges between 12 and 120. The total score is then converted to a 10–100 scale. The ACL-RSI has been reported to have good internal consistency (alpha 0.96), and inter-item correlations 0.69 (minimum 0.49, maximum 0.83) [29]. Divergent validity analysis showed significant differences between patients who returned to sport and patients who did not [29]. The ACL-RSI has been translated into several languages. In the Swedish version the presence of one underlying factor was confirmed [29], but when assessed with the Rasch method, the ACL-RSI showed multidimensionality and strong correlations between items [30]. 

The KOOS consists of 42 items scored on five subscales: pain, symptoms, activities of daily living (not used in this study), function in sports and recreation (Sports), and quality of life (QoL) [31]. All items are scored using a Likert scale, with five attainable answers from 0, extremely negative, to 4, extremely positive. Each subscale is analyzed separately with scores from 0 to 100: severe to no symptoms [32]. The KOOS has a reported ICC of 0.85–0.9 for test-retest reliability [33], and there is no evidence for content validity [34]. The KOOS´s psychometric properties have been questioned [34], and when assessed with a Rasch analysis, criteria for one-dimensionality were respected only in two out of five subscales: Sports and QoL [35]. Specifically, it is important to acknowledge that the KOOS demonstrates suboptimal psychometric properties, especially in terms of its content and structural validity [34, 36]. In comparison to other PROs, the KOOS subcategories display the lowest responsiveness [35]. An investigation into the validity of using the KOOS for patients recovering from an ACL injury has been undertaken [37]. However, caution is advised in the interpretation of the results due to language barriers and limited statistical options. Taken together, the KOOS demonstrates acceptable reliability but notable limitations in content validity, structural validity, and responsiveness, particularly in populations recovering from ACL injury.

The 18-item version of the K-SES (K-SES_18_) [38] aims to evaluate knee-related self-efficacy. The K-SES_18_ is divided into two subscales: present (14 items) and future (4 items) knee-related self-efficacy. Each item is graded from 0 to 10, with 10 being the most positive response, that is, the greatest belief to successfully carry out a given physical task. The results from each item are added and divided by the number of items to generate a mean value for each subscale. For the shorter 18-item version, structural validity, internal consistency and construct validity were explored, but not content validity [38]. Results suggest high alpha for the future subscale (0.81–0.91), and very high alpha (0.93–0.96) for the present subscale, two factors with eigenvalues ≥ 1, and good construct validity [38]. When assessed with a Rasch analysis, the K-SES_18_ showed strong reliability but ceiling effects, disordered thresholds, and item misfit, which challenge assumptions of content and construct validity, as well as unidimensionality [39]. 

The Tegner aims to assess the level of knee-strenuous activity [23]. The original scale ranges from 0 to 10, however, Project ACL uses a modified version which starts from level 1, where level 0 (disability due to sick leave) was removed. Higher level of Tegner indicate higher levels of knee-strenuous activity. From level 6 only sport activities are represented. Thus, if a patient rates Tegner 6 or higher it is assumed the patient is active within sports. The Tegner showed acceptable test-retest reliability (ICC = 0.8) [40], acceptable floor and ceiling effects, and showed to be responsive to change during rehabilitation in patients treated with ACL reconstruction [40]. 

### Study procedure

In this study, RTS was defined as return to an activity level graded 6 or greater on the Tegner. Female patients who returned to sport within 12 months of their primary ACL-R were divided into two groups based on whether they sustained a second ACL injury within 24 months. A second ACL injury was defined as either an ipsilateral graft rupture or a contralateral ACL injury after primary ACL-R, irrespective of the timing of return to sport.

Outcomes of interests were then compared between females who suffered a second ACL injury and female patients who “survived” 24 months after ACL-R without suffering a second ACL injury.

The data obtained from the SKLR consisted of demographics including sex, age, height, weight, BMI, time from injury to surgery, concomitant injuries and surgical procedures, and graft type for ACL-R. Data queried from Project ACL consisted of time of RTS, second ACL injury (ipsilateral or contralateral), time from surgery to second ACL injury, and results of muscle function tests and PROs. Incidence of a second ipsilateral or contralateral ACL injury within the follow-up period after ACL-R was registered as a dichotomous variable (yes/no).

Data from muscle function tests and PROs from the follow-up in which patients reported to RTS (defined as return to Tegner level ≥ 6), which could be the 4- 8- and 12- months follow-up after ACL-R, was used for analysis. Test results were not used to clear patients to RTS. Patients were then followed up to the 24 months after primary ACL-R to screen for the occurrence of a second ACL injury.

### Outcomes

The outcomes of interest in this study were the comparisons in response to PROs and results of muscle function tests between females who RTS within 12 months of ACL-R and who suffered or did not suffer a second ACL injury within 24 months of ACL-R. All outcomes were prespecified and analyzed descriptively. To aid in the interpretation of the analysis, PASS and MCID thresholds for PROs were used, but no responder-based analyses were performed.

### Statistical analysis

Descriptive statistics for continuous variables were presented as mean and standard deviation (SD), and median with minimum and maximum values, while categorical variables were presented as count (n) and proportion (%). In the comparisons between female patients who suffered a second ACL injury within 24 months of ACL-R and those who did not, between-group comparisons were performed using Fisher’s non-parametric permutation test for continuous variables. For dichotomous variables, Fisher’s exact test was used. For non-ordered categorical variables, the chi-square exact test was applied, and the 95% confidence interval (CI) for the difference and effect sizes (ES) were calculated. The 95% CIs were calculated using Fisher’s non-parametric permutation test. For dichotomous variables, the 95% CI was based on unconditional exact limits; if these could not be calculated, asymptotic Wald confidence limits with continuity correction were used instead. Effect sizes were calculated as the absolute mean difference divided by the pooled standard deviation. The Cohen´s d was interpreted according to the following values: 0.00 < 0.09 = negligible; 0.10 < 0.29 = small; 0.30 < 0.49 = medium; 0.50 or more = large [41]. All statistical analyses were performed using SAS (SAS Institute Inc., Cary, NC, USA), and the significance level was set to 0.05.

## Results

**Fig. 1:**
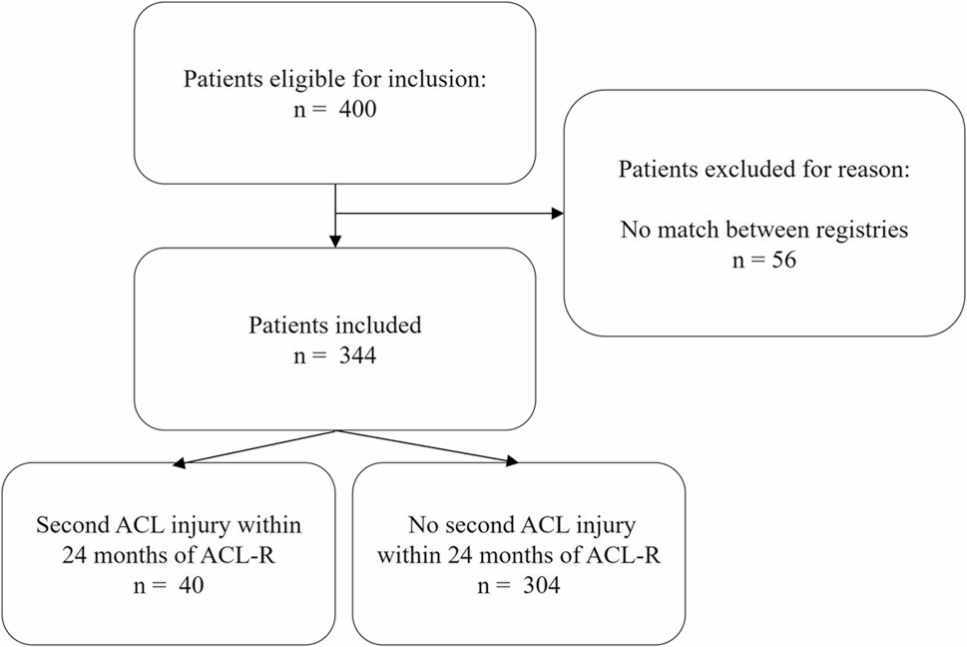
Flowchart of inclusion. ACL = anterior cruciate ligament; ACL-R = anterior cruciate ligament reconstruction; n = number; SKLR = Swedish knee ligament registry; Tegner = Tegner activity scale

A total of 344 female patients who RTS within 12 months of ACL-R were included in this study, of which 40 (11%) suffered a second ACL injury within 24 months of ACL-R (Fig. 1). Among the 40 female patients who sustained a second ACL injury within 24 months, 26 (65%) had an ipsilateral graft rupture and 14 (35%) sustained a contralateral ACL injury.

Effect sizes were interpreted according to conventional thresholds, where values of approximately 0.2, 0.5, and 0.8 represent small, medium, and large effects, respectively [41]. For example, an effect size of 0.3 would indicate a small-to-moderate difference between groups, whereas values close to zero indicate negligible differences.

### Demographics

Female patients who did not suffer a second ACL injury within 24 months of ACL-R received surgery on average 2.5 months later compared with females who suffered a second ACL injury (*p* = 0.015; ES = 0.25). The average time to RTS was 10 months, without significant difference between groups (Table 1). No other significant differences in demographic variables were observed between female patients who suffered a second ACL injury and female patients who did not (Table 1).

**Table 1:** Comparison between demographics for included female patients who suffered a second ACL injury and female patients who did not

	Total *n* = 344	No second ACL injury *n* = 304	Second ACL injury *n* = 40	*p*-value	Difference between groups; mean (95% CI)	Effect size
Age at ACL-*R*, years, mean (SD)	21.4 (4.9)	21.5 (5.0)	20.1 (3.6)	0.062	1.47 (-0.06; 3.16)	0.30
Height, cm, mean (SD)	168.4 (6.2)	168.3 (6.3)	168.7 (4.8)	0.74	-0.35 (-2.36; 1.64)	0.06
Weight, kg, mean (SD)	65.2 (8.4)	65.3 (8.5)	64.6 (7.6)	0.66	0.68 (-2.05; 3.52)	0.08
BMI, mean (SD)	23.0 (2.4)	23.0 (2.5)	22.7 (2.2)	0.40	0.34 (-0.44; 1.14)	0.14
Time to RTS, months, mean (SD)	10.0 (3.1)	10.1 (3.1)	9.2 (3.4)	0.11	0.77 (-0.19; 1.71)	0.27
Pre-injury Tegner activity level, n (%)						
6	11 (3.2%)	11 (3.6%)	0 (0%)	0.63		
7	43 (12.5%)	39 (12.8%)	4 (10%)			
8	108 (31.4%)	90 (29.6%)	18 (45%)			
9	119 (34.6%)	111 (36.5%)	8 (20%)			
10	63 (18.3%)	53 (17.4%)	10 (25%)			
Time from ACL injury to ACL-R, months, mean (SD)	5.6 (10.4)	5.9 (10.6)	3.3 (2.6)	0.015	2.54 (0.26; 6.70)	0.25
Injury activity at primary ACL injury, n (%)						
Alpine skiing/telemark	34 (9.9%)	34 (11.2%)	0 (0.0%)	0.23		
Pivoting sport e.g. football, floorball, handball, basketball, combat sports	293 (85.2%)	254 (83.6%)	39 (97.5%)			
Non-pivoting sport	4 (1.2%)	4 (1.3%)	0 (0.0%)			
Other	1 (0.3%)	1 (0.3%)	0 (0.0%)			
missing	0	0	0			
Graft type, n (%)						
Hamstring tendon	249 (78.8%)	216 (77.7%)	33 (86.8%)	0.056		
Patellar tendon	65 (20.6%)	61 (21.9%)	4 (10.5%)			
Allograft	2 (0.6%)	1 (0.4%)	1 (2.6%)			
Missing	28	26	2			
Meniscal status						
Concomitant meniscus injury, yes, n (%)	143 (41.6%)	129 (42.4%)	14 (35%)	0.47		
Meniscus repair, yes, n (%)	37 (10.8%)	32 (10.5%)	5 (12.5%)	0.87		
Meniscectomy, yes, n (%)	85 (24.7%)	74 (24.3%)	11 (27.5%)	0.79		

*ACL*  anterior cruciate ligament, *ACL-R*  anterior cruciate ligament reconstruction, *cm*  centimeters, *CI*  confidence interval, *kg*  kilograms, *n*  number, *RTS*  return to sport

### Patient reported outcomes

Female patients who suffered a second ACL injury within 24 months of ACL-R scored higher on KOOS Symptoms (83.8 vs. 78.5, mean difference 5.33, 95% CI 0.68–10.31, ES = 0.37, *p* = 0.024) compared to females who did not suffer a second ACL injury (Table 2).

**Table 2:** Results from patient reported outcomes at time of RTS between female patients who suffered a second ACL injury and those who did not.

	No second ACL injury *n* = 304	Second ACL injury *n* = 40	*p*-value	Difference between groups; mean (95% CI)	Effect size
KOOS Pain	88.1 (9.6)	90.3 (9.4)	0.16	-2.22 (-5.51; 0.86)	0.23
KOOS Symptoms	78.5 (14.7)	83.8 (12.3)	0.024	-5.33 (-10.31; -0.68)	0.37
KOOS Sports	72.6 (19.6)	75.4 (20.8)	0.40	-2.80 (-9.45; 3.49)	0.14
KOOS QoL	60.1 (16.4)	61.0 (19.8)	0.76	-0.869 (-6.486; 4.771)	0.05
K-SES_18_ Present	8.3 (1.4)	8.4 (1.6)	0.83	-0.067 (-0.583; 0.397)	0.05
K-SES_18_ Future	7.7 (1.6)	7.9 (1.3)	0.60	-0.143 (-0.673; 0.363)	0.09
ACL-RSI	62.2 (19.1)	66.4 (19.7)	0.27	-4.22 (-11.87; 3.23)	0.22

*ACL*  anterior cruciate ligament, *ACL-RSI*  anterior cruciate ligament return to sport after injury scale, *CI*  confidence interval, *KOOS*  knee injury and osteoarthritis outcome score, *K-SES*  knee self-efficacy scale, *n*  number, *QoL*  quality of life, *RTS*  return to sport

### Muscle function tests

There was no significant difference in the LSI of muscle function tests, i.e. isokinetic knee flexion and extension, as well as vertical hop, hop for distance and side hop, at time of RTS between female patients who suffered a second ACL injury within 24 months of ACL-R and female patients who did not (Table 3).

**Table 3:** Results from muscle function tests at time of RTS between female patients who suffered a second ACL injury and those who did not.

LSI (%)	No second ACL injury *n* = 304	Second ACL injury *n* = 40	*p*-value	Difference between groups; mean (95% CI)	Effect size
Knee extension	91.5 (12.3)	94.9 (9.2)	0.12	-3.40 (-7.74; 0.83)	0.28
Knee flexion	98.3 (11.3)	97.9 (9.0)	0.83	0.443 (-3.498; 4.467)	0.04
Vertical hop	88.4 (15.1)	92.9 (16.2)	0.14	-4.48 (-10.21; 1.47)	0.29
Hop for distance	94.7 (9.3)	96.2 (10.0)	0.39	-1.53 (-5.12; 2.07)	0.16
Side hop	95.1 (17.3)	96.5 (18.0)	0.68	-1.40 (-8.12; 5.41)	0.08

*ACL*  anterior cruciate ligament, *CI*  confidence interval, *n*  number, *LSI*  limb symmetry index, *RTS*  return to sport

## Discussion

The main finding of this study was that, among female patients who returned to sport within 12 months of their primary ACL-R, there were no differences in psychological response (i.e., knee self-efficacy, emotion, confidence, and risk appraisal) or muscle function between those who sustained a second ACL injury within 24 months and those who did not. Females who sustained a second ACL injury reported somewhat higher self-reported knee function in terms of knee-related symptoms compared to female patients without a second ACL injury. Together, these findings add to existing evidence by showing that, in female patients returning to sport within 12 months after ACL-R, commonly used psychological and muscle function measures at return to sport do not clearly distinguish those who will go on to sustain a second ACL injury from those who will not. Importantly, the absence of between-group differences at RTS should not be interpreted as evidence of equal reinjury risk, but rather as an indication that commonly used psychological and muscle function measures at a single time point may be insufficient for risk stratification.

Female patients suffer a second ACL injury (ipsilateral or contralateral) to a greater extent than male patients: 22.8% vs. 20.3% within 24 months of RTS [42]. Registry data from Sweden further support that the risk of a contralateral ACL-R is significantly greater in female patients, with adolescent females being particularly susceptible [43]. Some studies suggest that both male and female patients who go on to suffer a second ACL injury may paradoxically report higher self-reported knee function and greater psychological status than patients who “survive” [16, 44, 45]. These higher self-reported scores may reflect a mismatch between perceived status and actual status, where patients feel overly confident and ready, but may not have adequately restored neuromuscular control or movement quality. In fact, impaired neuromuscular control and suboptimal movement quality, such as excessive dynamic knee valgus, delayed hamstring activation, or asymmetrical load distribution, have been linked to a higher risk of primary and secondary ACL injuries [46]. In our study, we did not observe any differences in the KOOS Pain and Sports, QoL, K-SES_18_ or ACL-RSI between females who sustained a second ACL injury and those who did not. The absence of differences in these PROs may partly reflect limitations in the sensitivity of current instruments to detect subtle deficits in psychological variables or sport-specific function. Also, the absence of differences may indicate that psychological and functional profiles do not differ meaningfully between groups at the time of RTS. One possible explanation is that female patients at higher risk of reinjury may not experience or report pronounced symptoms or doubts despite incomplete recovery, leading to similar self-reported profiles at RTS [44, 45]. In addition, psychological factors related to RTS may operate differently across individuals and over time, which could further obscure between-group differences at a single assessment point [47, 48]. 

Furthermore, patients’ self-reported psychological and physical outcomes vary over time [25, 47, 48], and the timing of assessment likely influences the observed association, or lack of, with a second ACL injury. In this study, no clear psychological response, self-reported knee function and muscle function variable distinguished female patients who sustained a second injury from those who did not. This absence of a differences may itself be clinically important: previous studies have shown that both higher and lower scores on psychological PROs can be associated with an increased risk of a second ACL injury [16, 44, 45]. Thus, relying solely on self-reported outcomes may fail to identify high-risk individuals, as they do not present a consistent psychological or functional profile at RTS. For clinicians, this highlights the importance of combining PROs with objective neuromuscular assessments and individualized risk profiling to guide RTS decisions and potentially reduce the occurrence of a second ACL injury. Future research including other design and metrics is warranted to identify modifiable factors that could help detect female patients at higher risk of a second ACL injury. Also, to stratify patients based on timing of return to sport and to incorporate information on sport exposure, training intensity, and activity volume may help clarify whether distinct psychological or functional profiles emerge over time. In addition, studies with larger numbers of second ACL injuries could apply multivariable or longitudinal modelling approaches to examine how psychological, physical, and anatomical factors jointly contribute to reinjury risk.

In our results we could observe a difference in self-reported knee function with a moderate effect size, where female patients who sustained a second ACL injury reported superior outcomes in terms of knee-related symptoms compared to female patients who did not suffer a second ACL injury. To aid in the interpretation of results in the PROs, scores can be assessed with the Patient Acceptable Symptom State (PASS), and the Minimum Clinically Important Difference (MCID). The MCID for KOOS has been reported to be 11.9 for symptoms [49], while PASS was 57.1 for the KOOS symptoms [50]. Accordingly, the mean difference for KOOS symptoms was below MCID values indicating that the observed differences was not of a magnitude considered clinically important at the group level. Further, PASS values for both females who did and did not suffer a second ACL injury were above acceptable values. The result that female patients who sustained a second ACL injury reported fewer symptoms may reflect a misalignment between perceived recovery and actual tissue readiness or movement competence [51]. This paradox could indicate that reduced symptom burden may inadvertently contribute to a premature return to high-risk activity. For clinicians, these findings reinforce the importance of not equating absence of symptoms with full functional recovery. However, given the risk of type I error, and the values below the MCID, differences in self-reported knee symptoms should be interpreted cautiously.

In our study, we observed no significant differences in LSI of muscle function tests between female patients who sustained a second ACL injury and those who did not. This aligns with existing literature questioning the value of LSI to assess the risk of a subsequent ACL injury [52]. Importantly, this does not imply that muscle strength or functional tests lack clinical value, but rather that their role may be complementary rather than sufficient when used in isolation for reinjury risk stratification. Muscle strength and functional testing remain central components of RTS assessments and have demonstrated value in evaluating rehabilitation progress and usefulness to clear patients for sport participation [15, 53]. However, these findings suggest that LSI, when used in isolation, may be insufficient to identify patients at increased risk of a second ACL injury. While many patients achieve acceptable LSI thresholds post-rehabilitation (≥ 90% LSI), this is not necessarily associated with a reduced risk of reinjury [53]. The use of LSI alone may not capture the complexity of neuromuscular recovery and the multifactorial nature of ACL injury risk. A more comprehensive assessment, that incorporated both different objective measures of neuromuscular function and different self-reported evaluations of psychological variables are recommended to better inform decision making in the RTS process and potentially mitigate the risk of a second ACL injury.

In demographics, female patients who did not sustain a second ACL injury had a slightly longer time from injury to surgery compared with those who sustained a second injury. Although this difference reached statistical significance, the effect size was small and the clinical relevance is uncertain. Evidence on the impact of timing to ACL reconstruction is mixed: some studies report better patient-reported outcomes with early reconstruction compared with delayed crossover from non-operative management [54], but overall systematic reviews do not conclusively favor early over delayed reconstruction for all functional outcomes, and longer delays have been associated with greater risk of concomitant cartilage and meniscal pathology rather than reinjury per se [55]. Given the observational design and the small effect size, this finding should be interpreted cautiously and may represent a marker of patient selection rather than a causal factor.

### Limitations

This study has some limitations which infers caution in the interpretation of our results. First, while statistically significant differences were observed in some PROs, there is a risk of type I error due to multiple comparisons and the relatively small number of patients who sustained a second ACL injury. The limited number of second ACL injuries reduces statistical power to detect small between-group differences, and smaller effects cannot be ruled out. To mitigate the risk of type I error, effect sizes and mean differences with 95% CI were provided. Given the exploratory nature of the study and that several of the outcome measures analyzed are conceptually and statistically correlated, including subscales derived from the same questionnaire and related muscle function tests, violates the assumption of independence underlying Bonferroni-type corrections. Accordingly, no formal adjustment for multiple comparisons was applied; results were interpreted based on effect sizes, confidence intervals, and overall consistency of findings. Second, although we defined RTS as reporting Tegner level ≥ 6, this definition does not provide information about the actual type of sport performed, or volume, frequency, or intensity of sport participation. Consequently, the true exposure to sport-specific demands may have varied substantially between patients classified as having RTS. Further, structural anatomical factors such as tibial slope, hypermobility and femoral intercondylar notch morphology, which are known risk factors for a second ACL injury [56–58], were not taken into account. Specifically, females tend to have relatively narrower notch dimensions compared with males, and reduced notch width indices have been associated with increased ACL injury risk [6]. Fourth, while the LSI remains a widely used metric to assess recovery of muscle function, it has important limitations. The LSI assumes that the uninjured limb is a valid comparator, which may not be the case due to bilateral deficits or compensatory movement patterns [53]. Fifth, although the use of large-scale registry data increases external validity, it also limits the depth of biomechanical and contextual data that could be obtained, such as neuromuscular control, joint loading, or rehabilitation adherence. Finally, residual confounding related to age, graft choice, sport-specific exposure, and unmeasured neuromuscular or movement-quality factors cannot be excluded and may have influenced the observed associations (or lack thereof).

## Conclusion

No differences were observed in psychological response, that is, self-reported knee self-efficacy and confidence, emotion, risk appraisal, or muscle function between females who sustained a second ACL injury within 24 months and those who did not, in a cohort of females who returned to sport within 12 months after ACL-R. However, females who experienced a second injury reported slightly fewer symptoms at RTS, although not above established thresholds for clinical relevance. Clinically, these findings indicate that the absence of symptoms or psychological concerns at return to sport should not be interpreted as low reinjury risk, and that return-to-sport decisions in female patients may require assessment approaches beyond single time-point patient-reported outcomes or muscle function tests.

## Data Availability

The datasets used and/or analysed during the current study are available from the corresponding author on reasonable request.

## Abbreviations

ACL: Anterior Cruciate Ligament
ACL-R: Anterior Cruciate Ligament Reconstruction
ACL-RSI: ACL-Return to Sport after Injury scale
CI: Confidence Interval
cm: Centimeters
ES: Effect Size
ICC: Intraclass Correlation Coefficient
KOOS: Knee Injury and Osteoarthritis Outcome Score
K-SES18: Knee Self Efficacy Scale18 Items Version
LSI: Limb Symmetry Index
PROs: Patient Reported Outcomes
RECORD: Reporting of studies conducted using observational routinely-collected health data
RTS: Return to Sport
SD: Standard Deviation
SKLR: Swedish Knee Ligament Registry
